# Lateral hypothalamic area high-frequency deep brain stimulation rescues memory decline in aged rat: behavioral, molecular, and electrophysiological study

**DOI:** 10.1007/s00424-024-03059-z

**Published:** 2025-01-21

**Authors:** Abdelaziz M. Hussein, Ahmed F. Abouelnaga, Walaa Obydah, Somaya Saad, Marwa Abass, Asmaa Yehia, Eman M. Ibrahim, Ahmed T. Ahmed, Osama A. Abulseoud

**Affiliations:** 1 Department of Medical Physiology, Faculty of Medicine, Mansoura University, Mansoura (35516), Egypt; 2 Department of Animal Behavior and Management, Faculty of Veterinary Medicine, Mansoura University, Mansoura, 35516 Egypt; 3 Department of Surgery, Anesthesiology, and Radiology, Faculty of Veterinary Medicine, Mansoura University, Mansoura, 35516 Egypt; 4 Department of Neuroscience, Graduate School of Biomedical Sciences, Mayo Clinic College of Medicine, Phoenix, AZ USA; 5 Department of Anatomic Pathology, Faculty of Medicine, Mansoura University, Mansoura, 35516 Egypt; 6 Menninger Department of Psychiatry and Behavioral Sciences, Baylor College of Medicine, Houston, TX 77030 USA; 7 Department of Psychiatry and Psychology, Mayo Clinic, Phoenix, AZ USA

**Keywords:** Aging, LFPs, LHA, CA1 hippocampus, DBS, Hsp70, BDNF, Synaptophysin, Nrf2, Oxidative stress

## Abstract

To examine the effect of DBS of the lateral hypothalamic area (LHA) on age-related memory changes, neuronal firing from CA1, oxidative stress, and the expression of Hsp70, BDNF, and synaptophysin. 72 male rats were randomly allocated into 6 equal groups: a) normal young group (8 W), b) sham young group, c) DBS young group, d) normal old group (24 months), e) sham old group and f) DBS old group. Memory tests (passive avoidance and Y maze), oxidative stress markers (MDA, catalase, and GSH) and expression of Nrf2, HO-1, Hsp70, BDNF, and synaptophysin were measured by the end of the experiment. Also, in vivo recording of the neuronal firing of the CA1 region in the hippocampus was done. Old rats show significant decline in memories, antioxidant genes (Nrf2 and HO-1), antioxidants (GSH and catalase), Hsp70, BDNF, and synaptophysin with significant increase in MDA in hippocampus (*p* < 0.05) and DBS for LHA caused a significant improvement in memories in old rats, with significant rise in fast gamma and theta waves in CA1 region in old rats (*p* < 0.05). This was associated with a significant increase in antioxidants (GSH and CAT), antioxidant genes (Nrf2, HO-1), Hsp70, BDNF, and synaptophysin with significant reduction in MDA in hippocampus (*p* < 0.05). DBS for LHA ameliorates the age-induced memory decline. This might be due to increase in fast gamma in CA1, attenuation of oxidative stress, upregulation of Nrf2, HO-1, Hsp70, BDNF, and synaptophysin in the hippocampus.

**Graphical Abstract:**

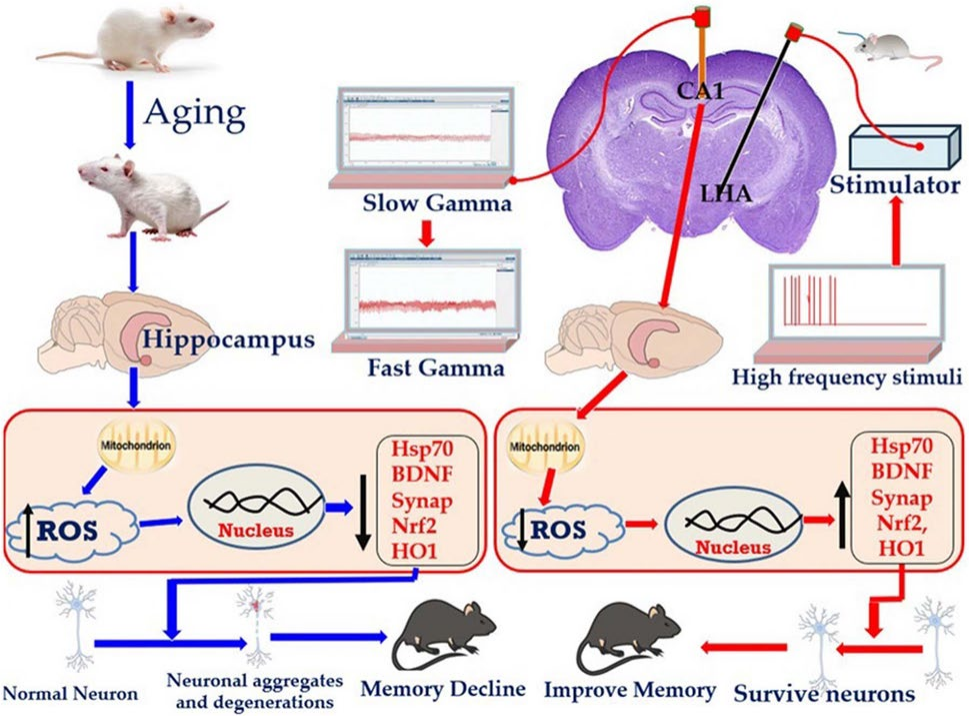

The effect of DBS of LHA on memory decline in aged rats. The process of aging results in degeneration of the hippocampus (critical region for memory and learning) via increasing the production of ROS which results in downregulation of Nrf2, HO1, Hsp70, BDNF and synaptophysin leading neuronal aggregates, degeneration and memory decline. On the other hand, DBS of LHA by high frequent currents survive the neurons of hippocampus via reduction of ROS production and upregulation of antioxidant genes (Nrf2/HO1), BDNF, synaptophysin and Hsp70 leading to improvement of memory decline. DBS = deep brain stimulation, LHA = lateral hypothalamic area, ROS = reactive oxygen species, Nrf2 = nuclear erythroid related factor, HO-1 = heme oxygenase-1, BDNF = brain derived neurotrophic factor, Hsp70 = heat shock protein 70. ↑ = increase, ↓ = decrease. Red arrows indicate the process of aging, while blue arrows indicate the process of DBS.

## Introduction

Aging is the process through which an organism's ability to function declines over time. ***Zhao *et al*.*** [93]reported that the aging process is accompanied by physical and cognitive deterioration, as well as a higher risk of neurodegenerative disorders. ***Lee *et al*.*** [44]concluded that aging is a significant risk factor for neurodegenerative disorders, which are marked by increased numbers of senescent cells in the brain, protein aggregation, and central nervous system atrophy. Neurodegeneration, which is primarily sporadic and manifests at an older age than inherited neurodegeneration, is commonly associated with Alzheimer’s disease (AD) and characterized by reduced synaptic connections and progressive neuronal loss [93, 40]. Mechanisms underlying the process of aging-induced neurodegeneration are complex and still not fully elucidated. It might include enhanced oxidative stress in brain areas [38], downregulation of the antioxidant genes, such as nuclear factor erythroid 2–related factor 2 (Nrf2) [61] and neuroprotective heat shock proteins (Hsp) e.g. Hsp70 that protect against neuronal degeneration [26, 7], impaired neurogenesis (BDNF) and reduced synaptic connections [40, 94] and electrophysiological changes in the regions involved in memory formation and consolidation.

The lateral hypothalamic area (LHA) is a critical brain region for optimal functionality through coordinating energy expenditure with sleep–wake cycle [56, 67]. ***Ishii and Iadecola***^36^]***,*** suggested that the hypothalamus is involved in the early pathology of dementia. Indeed, postmortem studies in patient with Alzheimer disease (AD) reported the presence of plaques and tangles in the hypothalamus [90] and neuroimaging studies found reduced glucose metabolism [22]. Moreover, disruptions of certain hypothalamic functions such as circadian rhythm [60] and sleep–wake cycle [88] are evident in patients with dementia. In addition, a case report shown that hypothalamic stimulation in a patient with morbid obesity modulated limbic activity and improved certain memory functions [69]. Whether hypothalamic dysfunction, according to these studies, contribute directly to cognitive decline or indirectly through the hippocampus remains under investigation. Strong projections connect the hippocampus and hypothalamus. It has been shown that, there is widespread projections from the LHA span the entire cerebral cortical mantle including the hippocampal formation and the most prominent single-region source of input to the LHA is the hippocampal formation specially CA1 and subiculum. Similarly, fibers originating in the LHA terminate in the fornix, and CA1 regions of the hippocampus [27]. Additionally, memory impairment can result from damage to the fornix, which is intimately connected to the lateral hypothalamus [84, 10].

While most neurodegenerative disorders have no known cure, there are a number of medicines and interventions that can help control the symptoms and improve the course of the disease and the quality of life for affected individuals [64]. FDA approved the use of deep brain stimulation (DBS) for management of several neurodegenerative disorders such as Parkinson's disease, dystonia, essential tremor, epilepsy, and obsessive–compulsive disorder [54]. A number of previous studies demonstrated beneficial effects for the DBS in some brain regions which are involved in the memory process and closely related to the LHA such as forniceal area on memory decline [82, 45, 30, 70, 53]. However, no study, up to the best of our knowledge, examine the effects of DBS of LHA on memory impairment. Therefore, in the current study, we hypothesized that age-related memory impairment is due, at least in part, to failure of lateral hypothalamic integrative function and DBS for LHA could improve this decline in memory functions. To test this hypothesis, we propose to compare the performance in memory tasks between young and aged rats and apply high frequency stimulations to the lateral hypothalamic area to enhance memory and reverse memory task impairment in young and aged rats, respectively. Furthermore, we investigated the underlying mechanism through in-vivo electrophysiological recordings in the CA1 region of the hippocampus and molecular assays to quantify stimulation-associated changes in oxidative stress markers and Hsp70, BDNF and synaptic vesicle proteins such as synaptophysin in different hippocampal regions after LHA stimulation.

## Materials and methods

### Experimental animals

Seventy-two male Sprague–Dawley rats were enrolled in this study. Rats were housed at the Mansoura Medical Experimental Research Center (MERC), Mansoura Faculty of Medicine, Mansoura, Egypt. All experimental procedures and protocols were approved by our local institutional review board (IRB, code #RP.19.09.41), Mansoura Faculty of Medicine, Mansoura, Egypt. Rats were individually housed in standard cages of polypropylene (48.5 cm length 33 cm width 21 cm height) with ad libitum access to food and water. The animal colony was maintained under controlled temp. (about 24–26 °C), humidity (60–70%), and 12 h. dark/light cycle (lights-off at 7.0 AM). All cages have been enriched by sawdust as a bedding material. Male rats were chosen in the current study to avoid the effect of the estrous cycles of females on the behavioral and physiological profiles [80, 1].

### Experimental design:

Rats were randomly subdivided into 2 groups (each 36 rats).

A) ***Young rat group (8-week-old and 130—150 g body weight)*** included 3 subgroups (each 12 rats).
  i. A young control group.
  ii. A young sham group that underwent a sham operation.
  iii. A young DBS group that underwent LHA DBS.
B) ***Old rat group (24-month-old and 450—490 g body weight)*** included 3 subgroups (each 12 rats).
  i. An old control group.
  ii. an old sham group that underwent a sham operation.
  iii. an old DBS group that underwent LHA DBS.

### Surgical implantation of stimulating and recording electrodes

We used intraperitoneal (IP) medetomidine (0.4 mg/kg, Domitor 1, Pfizer, Seixal, Portugal) and ketamine (50 mg/kg, Ketamax 5%, Troikaa, Gujarat, India) to anesthetize the rats [95, 23]. To avoid corneal ulceration, an eye cream was applied to both corneas. Rats' body temperature was kept between 37.5 and 38.5 °C using a heating pad. During surgery, we secured the rat in the digital stereotaxic frame instrument (model # 68,025, RWD life sciences, China) and leveled the skull between the bregma and lambda. Then, we implanted a unilateral bipolar stimulating electrode (#MS303/1-B/SPC twisted stainless steel, outer diameter 250 μm, Plastics One, USA) in the LHA (anteroposterior: − 2.28, mediolateral: ± 3.7, dorsoventral: − 8.5 mm from skull surface). Stimulating electrodes were implanted with a 14º angle to allow enough space to insert the recording electrode. Next, we implanted an ipsilateral recording electrode (#MS333/1-C/SPC stainless steel twisted insulated 3 channels electrodes, outer diameter 250 μm, Plastics One, USA) in the CA1 hippocampal region (anteroposterior: − 2.7, mediolateral: ± 1.0, dorsoventral: − 3.0 mm from skull surface at 0 angle). Electrodes were secured to the skull using dental cement and three screws. Rats were housed singly to prevent other rats from chewing the implant after the surgery. Animals were daily weighted and monitored for recovery over 5 days post-surgery. Animals with any neurological signs of brain damage, signs of sickness, wound infection, body weight loss, any signs of reduced well-being were excluded from the study. Also, any rat mortality was replaced by new ones because the start of our experiment was after the recovery period of the surgery. In one rat, an infection occurred at the implantation site with an accompanying dislocation of the electrode, so this rat was excluded for further studies. Also, two rats were dead during recovery period from brain infections and intracerebral bleeding had been replaced by new rats. Moreover, the unilateral stimulation and ipsilateral recording of LFPs were chosen in the current study because insertion of 4 electrodes in the rat’s brain prevent insertion the cables of recording and stimulation as well as we chose the right side because surgery is technically easier on the right side [33].

### Protocol of deep brain stimulation (DBS)

**Fig. 1:**
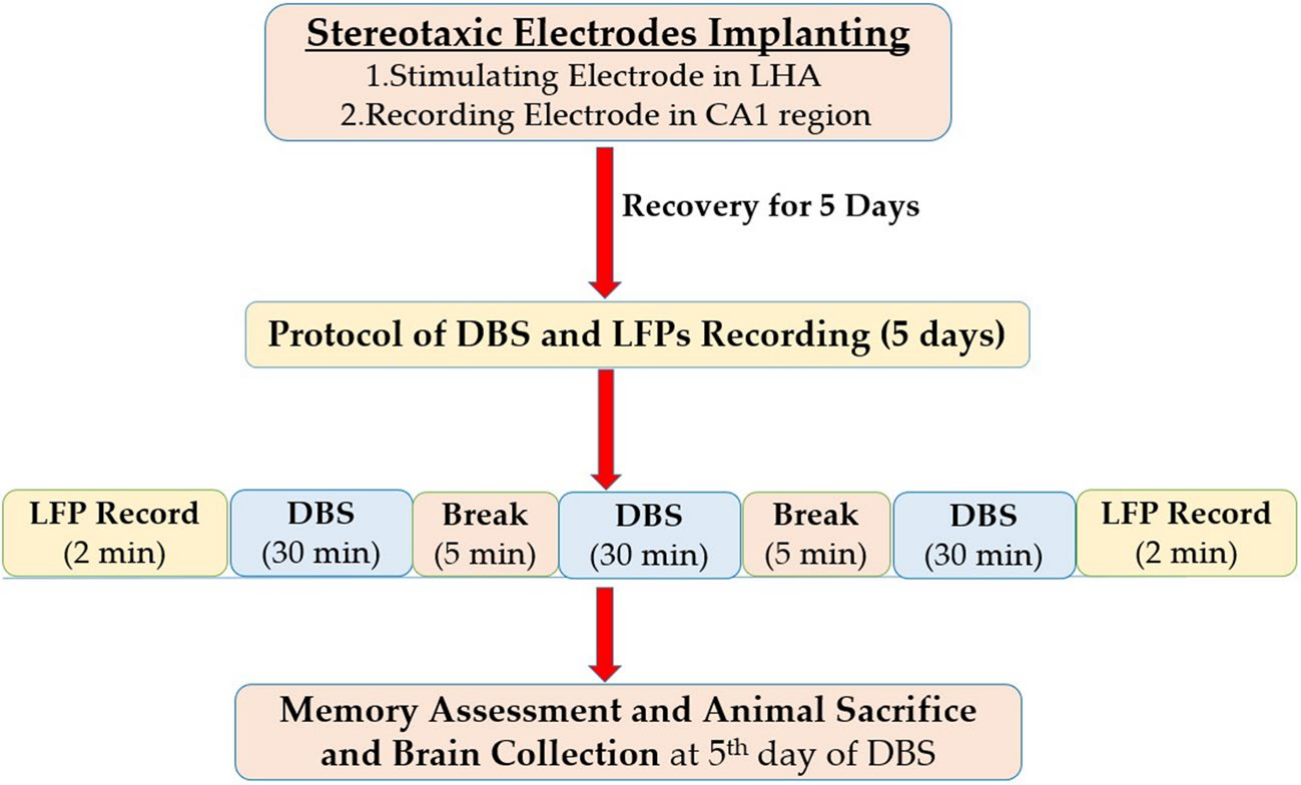
Study design

DBS was done by high-frequency (HF) electric currents (200 μA amplitude, pulse width of 100 μs, and frequency of 130 Hz) for 1.5 h. with a 5-min break every 30 min. for 5 consecutive days (Fig. 1). Stimuli were delivered using a stimulus isolator (FE180, ADInstruments, USA).

### In vivorecordings of hippocampal CA1 neuronal firing (LFPs)

Electrophysiological recording was done using the PowerLab 3/40 data acquisition system with an ML136 animal Bio Amplifier (ADInstruments, USA) and LabChart 7 software. The local field potentials (LFPs) from CA1 were recorded 2 min before DBS and immediately after the last DBS trial under the following parameters: (50 Hz notch filter, mains filters, EEG mode, sampling rate 1 k/s., range: 1 mV or less). The Fast Fourier Transform (FFT) analysis method was utilized to examine LFP recordings (power spectra are obtained by converting data from the time domain to the frequency domain using the FFT method). Five frequency bands were identified from the obtained averaged power spectra: delta (1–4 Hz), theta (4.1–8 Hz), alpha (8–12 Hz), beta (12–30 Hz), slow gamma (30–90 Hz), and fast gamma (> 90 Hz) [43]. For comparison purposes and to overcome the inter-individual variations, we normalized the band power by dividing its value by the total power of all bands. Normalization ratio (NR) = (power of each band/total power of all bands) × 100.

### Passive avoidance test (PAT) for retention working memory

The retention working memory of all rats was tested using the passive avoidance test (PAT) after DBS [6]. The retention memory of the rat is measured by its ability to remember a previously received electrical foot shock 24 h prior to the test. The passive avoidance apparatus is a wooden box with a larger (50 × 50 × 35 cm) and brightly illuminated room and a smaller (15 × 15 × 15 cm) dark compartment supplied with a grid floor that is connected to an electrical shock source. Both rooms are separated by a door that allows rats’ entry into the small, dark compartment. The retention memory test was divided into two sessions (1) exploration and learning, and (2) retention evaluation. During the first exploration session, while the door was open, each rat was placed in the illuminated large room and allowed to explore it freely for three five-minute trials at 30-min intervals. At the end of the 3rd trial, as the rat stepped into the dark compartment, the door was closed, and a single foot shock (50 Hz, 1.5 mA, for 1 s) was delivered directly through the grid floor. The rat was kept in the dark room for an additional 10 s before being returned to its home cage. The next day, a retention evaluation was performed by placing the rat into the large, illuminated room to record the time latency (in seconds) needed for the animal to step into the dark area.

### Y maze test for spatial memory

The effect of DBS on elderly rats' spatial memory was investigated using the Y-maze test. Each rat was placed in the middle of the Y-maze and allowed ten minutes to freely move around the three arms. Following every test, the device was cleaned with 70% ethanol to get rid of any olfactory cues, and the number and order of arm entries were recorded. The animals scored a point for an arm entry when all four of their paws were inside any of the Y-maze's arms. We calculated \ the percentage of spontaneous alternation (spontaneous alteration = total number of accurate alterations between the three arms/ (total number of arms entries-2) × 100) [41].

### Brain tissue harvesting

By the end of the experiments, 6 rats in each group were transcardially perfused with 150 ml of saline (0.9%), and their brains were harvested and stored at −80 ^◦^C for biochemical analysis of oxidative stress markers and gene expression for antioxidant genes (Nrf2 and heme oxygenase 1 (HO-1)). The remaining 6 rats were perfused transcardially with 50 ml of saline (0.9%) followed by 100 ml of 4% paraformaldehyde to do in situ fixation of the brain, then fixed with 4% paraformaldehyde for 24 h at 4 °C for histological and immunohistochemical examination.

### The accurate location of the stimulating electrode in the LHA and the recording electrode in the CA1

**Fig. 2:**
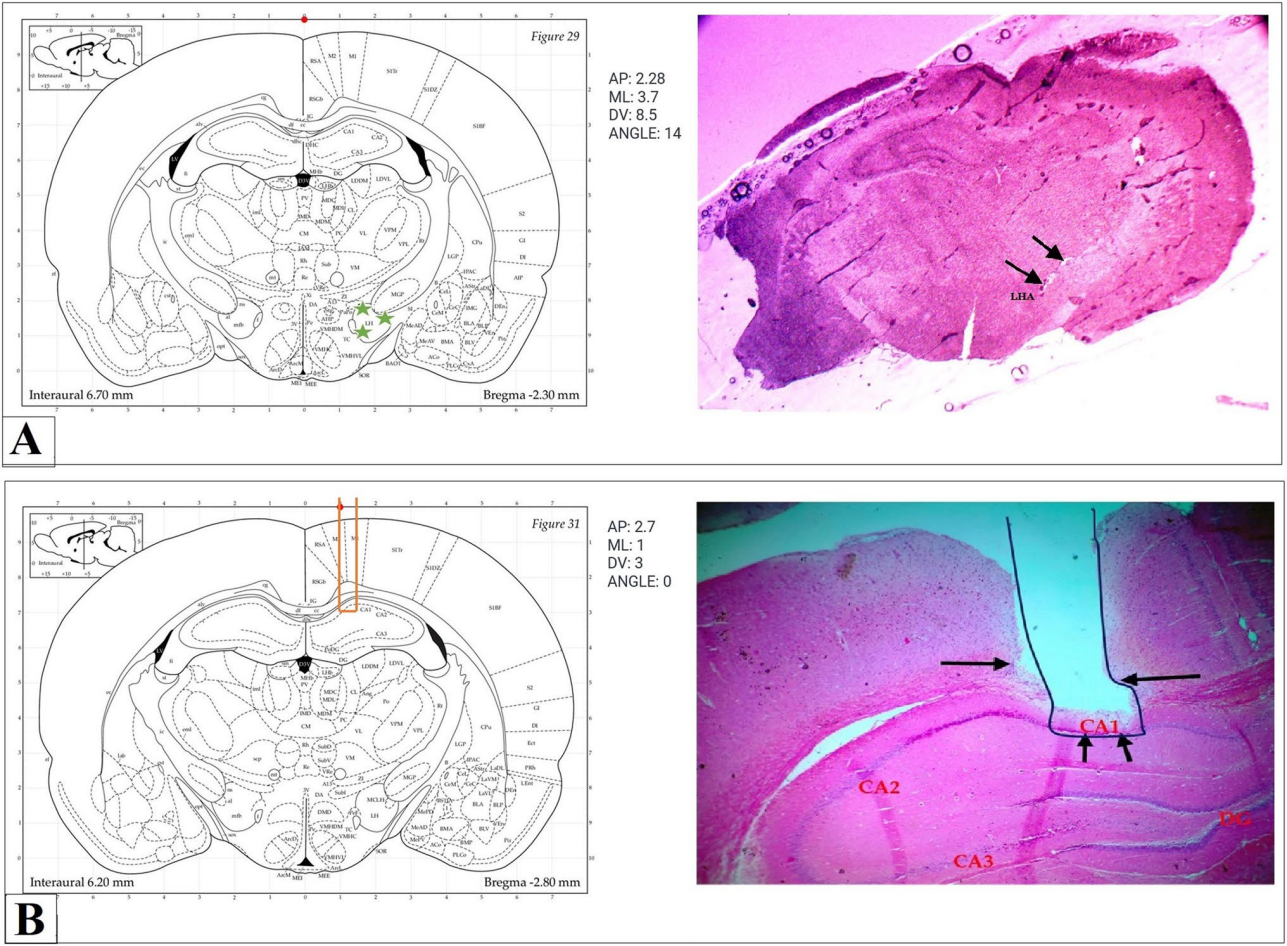
Tracing of the stimulating electrode in LHA (**A**) and Recording electrode in CA1 hippocampal region (**B**)

The destination of each electrode in the stimulating (LHA) and recording (CA1) regions was detected by doing either frozen sections or H&E staining for the brain after euthanasia. Figure 2A shows the place of the stimulating electrode in the LHA (frozen section), while Fig. 2B shows the place of the recording electrode in the CA1 region of the hippocampus (H&E section). Rats with misplaced electrodes were discarded from the experiment and replaced by new ones.

### Assessment of Nrf2 and HO-1 expression at the level of mRNA by real-time PCR

Using real-time PCR, we identified mRNA encoding for Nrf2, the antioxidant transcription factor, and HO-1 in the hippocampus. According to the manufacturer’s instructions (Sangon Biotech Co., Ltd. Shanghai, China), we isolated the total RNAfrom brain tissue specimens. We quantified RNA spectrophotometrically and determined its quality by agarose gel electrophoresis and ethidium bromide staining. cDNA was synthesized from 1 μg of total RNAand then buffered in 25 μL. Then, the 25 μL cDNA was diluted in a total volume of 100 μL. All details of gene (Nrf2, HO-1, and GAPDH) amplification and detection were mentioned in our previous work [75].

### Assay of oxidative stress markers (MDA, catalase activity, and GSH) in hippocampal tissues

Hippocampal regions of the brain were homogenized in 1–2 ml of cold phosphate buffered saline (50 mM) in EDTA (1 mM) at pH 7.5 and centrifuged at 4,000 rpm at 4 °C for 15 min. The colorimetric assay of these markers (MDA, GSH and CAT) was done using commercially available kits (Bio-Diagnostics, Dokki, Giza, Egypt) according to the manufacturer’s instructions.

### Histological examination and quantification of neuronal dystrophy in hippocampal regions

Fixed brains were embedded in paraffin, cut into 6-µm-thick coronal sections at − 3.0 mm AP, and stained with H&E for histopathological examination. Different hippocampal regions were examined and quantified using light microscopy by observers who were blinded to the experimental conditions. Hippocampal damage was assessed based on neuronal dystrophy in the CA1, CA3, and dentate gyrus (DG) regions. Neuronal dystrophy was considered in any neuron showing a shrunken appearance with eosinophilic cytoplasm and a dark pyknotic nucleus. Neurons without such changes were classified as normal. Scoring of the dysmorphic changes in these areas was done by calculating the i) total number of cells in high power field (HPF) of 6 fields, ii) total number of normal cells in the same fields, iii) total number of dysmorphic cells in the same fields, and iv) mean of total cells and dysmorphic cells in the 6 fields. The dysmorphic or dystrophic score was calculated by dividing the mean of dysmorphic cells by the mean of total cells in all six fields [35].

### Immunohistochemical examination for Hsp70, BDNF, and synaptophysin in the CA3 and CA1 regions and DG of the hippocampus

We obtained 6-µm-thick formalin-fixed paraffin- embedded sections for immunohistochemical examination. Prior to immunostaining, pH 6.0 citrate antigen retrieval (ZUC028-500, Zytomed, Germany) treatment was performed at 121 °C for 7 min. Antibodies used for immunohistochemical examination included Hsp70 (ab53496, dilution 1:200, Abcam), BDNF (ab108319, dilution 1:5000, Abcam), and synaptophysin (ab14692, dilution 1:100, Abcam). Stained slides were imaged with an OPTIKA camera (Leica Biosystems, USA). The expression of each marker was observed in CA1, CA3 and DG regions of hippocampus blindly by expert pathologists. The amount of expression for each marker was quantified using ImageJ (National Institutes of Health, USA) to measure the percentage area of expression in six regions of interest (ROI) and calculated their mean.

### Sample size

To ensure the minimum sample size required to be reasonably likely to detect the hypothesized effect. The sample size was calculated on G. power program version 3.1.9.7, as follows; by using ANOVA: fixed effects, special, main effects and interactions with effect size = 0.40 (large), and alpha level was set at 0.05 for power analyses of the test was 90%. The total sample size was calculated 68 rats and we added 4 rats (~ 5%) to make the total sample size 72 rats.

### Statistical analysis

The statistical analyses were done suing SPSS version 16.0. The homoscedasticity of the data was tested via Leven’s test and the data that show heteroscedasticity were processed using log transformation. Two-Way ANOVA test with Tukey’s posthoc test were used for multiple group comparisons for all tested variables except LFPs we used repeated measures ANOVA. Graphs were made by graph prism version 5.0.

## Results

### Effect of DBS on memory tests (PAT and Y maze) in young and old rats

**Fig. 3:**
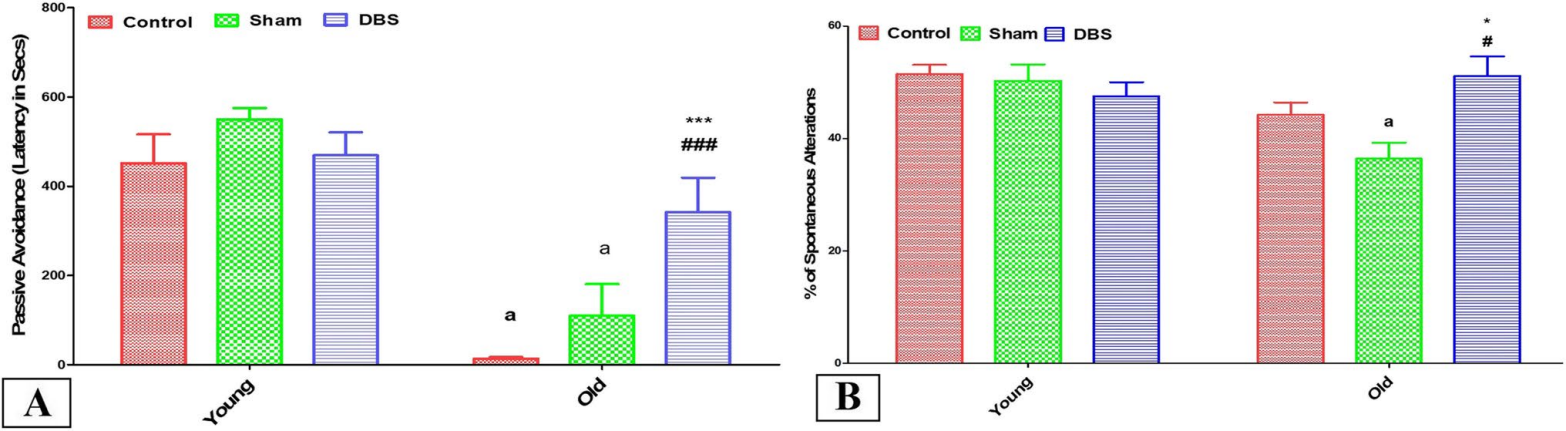
Memory tests including **A** = Y maze test (% of spontaneous alterations) and **B** = passive avoidance test (entry latency in seconds). Data are expressed as mean ± SEM. Two-way ANOVA with Tukey post hoc tests were used for comparing different groups of at different age times. ^a^ significant vs the corresponding young group, * significant vs the control group and #significant vs the sham group of the same age. *p < 0.05, ***p < 0.001, ^#^*p* < 0.05, ^###^*p* < 0.001. *DBS* deep brain stimulation

The working and spatial memories were tested using passive avoidance and Y maze tests, respectively. The control and sham old groups showed significant decrease in the entry latency time in PAT compared to their corresponding young subgroups (*p* < 0.0001) with no statistically significant difference between DBS young and old groups. Also, the latency time showed no statistically significant differences among all young subgroups (control, sham, and DBS). On the other hand, the latency time was significantly higher in the old DBS group compared to the old normal and sham groups (*p* < 0.001) (Fig. 3A). The percentage of spontaneous alternations in Y maze showed statistically significant reduction in old sham group compared to young sham group (p < 0.05) with non-significant reduction in old control group compared to young control group. On the other hand, % of spontaneous alternations in was significantly high in the old DBS group compared to old control and sham groups (*p* < 0.05). Moreover, there was no statistically significant differences among all young subgroups (Fig. 3B).

### Effect of DBS on LFPs in the CA1 hippocampal region of young and old rats

**Fig. 4:**
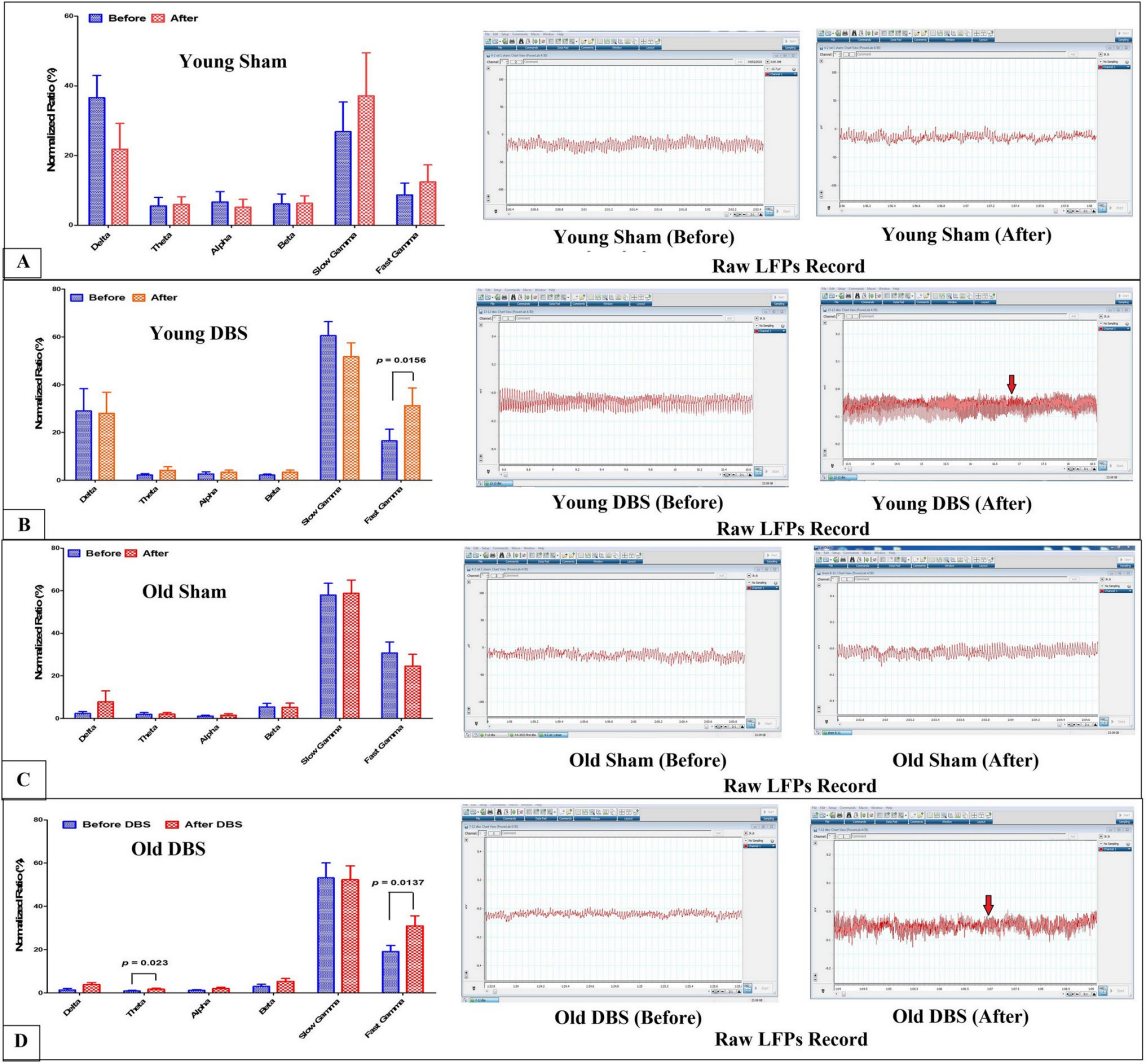
Normalized ratio of different waves of the local field potentials (LFPs) recorded from CA1 hippocampal region and raw recordings from; **A**) sham young group, **B**) young DBS group, **C**) old sham group and **D**) old DBS group before and after stimulation. Repeated measures ANOVA was used for comparing different waves before and after stimulation. Raw LFPs from each group before and after stimulation were presented. Red arrows indicate fast gamma waves in young DBS and old DBS groups

Young sham rats' normalized ratio (NR) of LFP waves recorded from CA1 revealed that the gamma and delta waves were the most prominent, with no significant difference seen between any of the waves in either recording (Fig. 4A).On the other hand, the NR values of LFPs waves from CA1 in the young DBS group did not show any significant difference in both recordings (before or after stimulation) except for the fast gamma wave, which showed a significantly higher value after DBS when compared to its value before DBS (*p* = 0.0156) (Fig. 4B). Similar results were observed in older groups. The NR of waves of LFPs recorded from CA1 in old sham rats did not show any significant difference between both recordings for all different waves (Fig. 4C). On the other hand, fast gamma waves in old DBS group demonstrated a significantly higher values after DBS when compared to its value before DBS (*p* = 0.0137) and theta waves demonstrated significantly higher values after DBS compared to its value before DBS (*p* = 0.023). However, the NR of other waves of LFPs recorded from CA1 did not show a significant difference between before and after values of any different waves in DBS group (Fig. 4D). Each group graph is accompanied by a sample of raw EEG or LFP recordings made from each group.

### Effects of DBS on oxidative stress markers (MDA, GSH and CAT) in young and old rats

The hippocampal MDA (a lipid oxidation marker) concentration levels show no statistical differences between the same groups of different ages. On the other hand, the sham groups (old and young groups) showed significantly higher levels compared to their control groups (*p* < 0.001). Furthermore, compared to sham groups, the hippocampal MDA concentration in DBS groups (both young and old) was considerably decreased (*p* < 0.001). Conversely, the GSH concentration in the hippocampal regions of the different old groups was significantly lower than that of their corresponding young groups (*p* < 0.001). Furthermore, the GSH concentration in the sham groups (young and old) was considerably lower than in their corresponding control groups (*p* < 0.001), and the GSH concentration in the DBS groups was higher than in their corresponding sham groups (*p* < 0.001). There were no statistically significant differences in the hippocampal CAT activity between the different groups of different ages (young and old). Additionally, the activity of CAT in sham groups was considerably lower than that of their corresponding control groups (*p* < 0.001). Additionally, Table 1 shows that the CAT activity in DBS groups was considerably higher than that of their corresponding sham groups (*p* < 0.001).

**Table 1:** The levels of Markers of oxidative stress (MDA, GSH and catalase) and expression of antioxidant genes (Nrf2 and HO-1) in hippocampal regions in different studied groups

	Control		Sham		DBS		Two-Way ANOVA
	Young	Old	Young	Old	Young	Old	*F *value, *P* value
MDA (nmol/g brain tissues)	23.01 ± 5.99	32.10 ± 2.772	105.8 ± 6.82^***^	85.79 ± 6.84 ***	97.50 ± 6.20 ***,###	76.93 ± 4.84 ***,###	• Age (0.15, 0.701) • DBS (293.17, < 0.001)
GSH (mmol/g brain tissues)	78.50 ± 3.34	56.19 ± 3.68 a	53.00 ± 23.96 **	43.22 ± 4.96 **	139.5 ± 26.88 ***,###	101.7 ± 3.37 a,***,###	• Age (21.61, < 0.001) • DBS (74.75, < 0.001)
Catalase (U/g brain tissues)	5.51 ± 0.52	5.53 ± 0.71	2.97 ± 0.51 ***	5.104 ± 0.18	5.88 ± 0.59 ###	4.08 ± 1.20 *,#	• Age (0.26, 0.614) • DBS (14.245, < 0.001)
Expression of Nrf2 at mRNA levels	1.03 ± 0.047	0.66 ± 0.032 a	1.79 ± 0.48 ***	0.59 ± 0.035 a	5.31 ± 0.66 ***,###	3.61 ± ^a, ***, ###^	• Age (62.094, < 0.001) • DBS (286.85, < 0.001)
Expression of HO-1 at mRNA levels	0.97 ± 0.015	0.68 ± 0.034 a	1.713 ± 0.16 *	0.703 ± 0.20 a	10.04 ± 0.34 ***,###	8.75 ± 0.62 a,***,###	• Age (70.917, < 0.001) • DBS (296.4, < 0.001)

Data are expressed as mean ± SEM. Two-way ANOVA with Tukey post hoc tests for comparing different groups of at different age times. ^a^ significant vs the corresponding young group, * significant vs the control group and #significant vs the sham group of the same age. *p < 0.05, **p < 0.01, ***p < 0.001, ^#^*p* < 0.05, ^##^*p* < 0.01, ^###^*p* < 0.001. DBS = deep brain stimulation

### Effect of DBS on the expression of antioxidant genes (Nrf2 and HO-1) in young and old rats

Older rats' hippocampal Nrf2 expression (in all old groups) was significantly lower than that of the rats of the corresponding young groups (*p* < 0.001). Also, the expression of Nrf2 was considerably higher in the young sham group compared to young control group (*p* < 0.001). Additionally, there was a significant increase in Nrf2 expression in the DBS groups (both young and old) as compared to the control and sham groups (both young and old) (*p* < 0.001). In addition, there was a significant increase in its expression in young DBS group than old DBS group (*p* < 0.001). Additionally, there was a significant decrease in HO-1 expression in all old groups compared to their corresponding young groups (*p* < 0.001). On the other hand, HO-1 expression was considerably higher in the young sham group compared to control young group (*p* < 0.001). Moreover, there was significant increase in its expression in DBS groups compared to their corresponding control and sham groups (*p* < 0.001) (Table 1).

### Effect of DBS on the neuronal damage (dystrophic score) in young and old rats in different hippocampal regions (CA1, CA3, and DG)

**Fig. 5:**
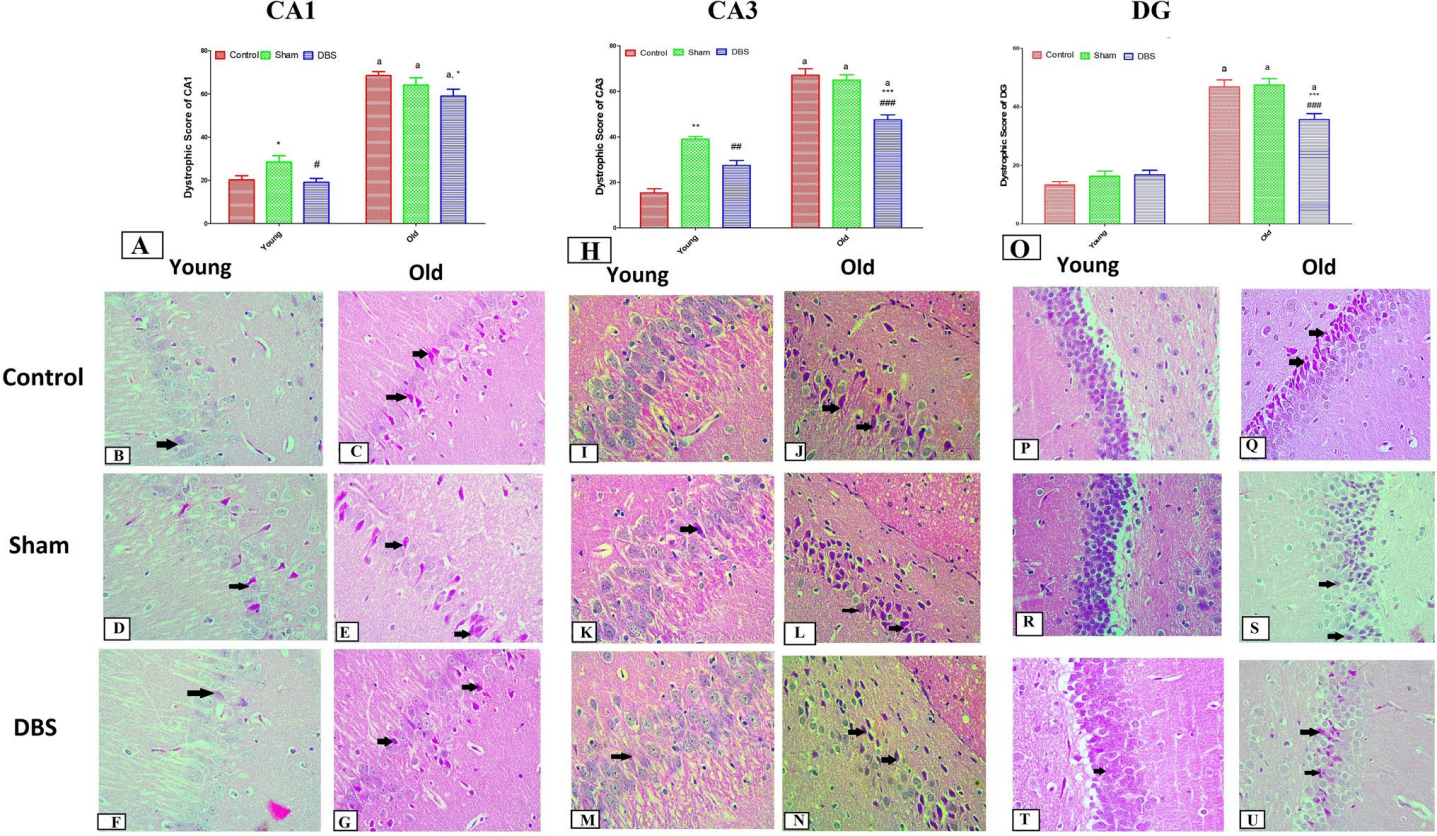
Histopathological examination from different groups in different hippocampal regions (CA1, CA3 and DG). Photomicrographs of dystrophic neurons (shrunken neurons with eosinophilic cytoplasm and a dark pyknotic nucleus) (black arrows) are shown in Fig. **A** along with the dystrophic score in the CA1 region of hippocampi from various young and old groups. Dystrophic neurons are few in the young control group (**B**), increased in the old control group (**C**), slightly increased in the young sham group (**D**), high in the old sham group (**E**), low in the young DBS group (**F**), and high in the old DBS group (**G**). Figure H show the dystrophic score from CA3 and photomicrographs from CA3 regions show no dystrophic neurons in the young control group (**I**), moderate in the old control group (**J**), few in the young sham group (**K**), high in the old sham group (**L**), and few in the young DBS group (**M**). Figure O shows the dystrophic score from DG and photomicrographs from DG regions show no dystrophic neurons in the young control group (**P**), moderate in the old control group (**Q**), few in the young sham group (**R**), high in the old sham group (**S**), and few in the young DBS group (**T**) and moderate in the old DBS (**U**). Two-way ANOVA with Tukey post hoc tests were used for comparing different groups of at different age times. ^a^ significant vs the corresponding young group, * significant vs the control group and #significant vs the sham group of the same age. *p < 0.05, **p < 0.01, ***p < 0.001, ^#^*p* < 0.05,^##^*p* < 0.01, ^###^*p* < 0.001. DBS = deep brain stimulation

In the CA1 region, the dystrophic score was significantly higher in old groups (control, sham, and DBS) compared to their corresponding young groups (*p* < 0.001). Additionally, there was a substantial decrease in the dystrophic damage score in young DBS compared to young sham group and in old DBS group compared to old control group (*p* < 0.001) (Fig. 5A). The CA1 region displayed moderate neuronal dystrophic alterations in photomicrographs taken from young groups (control, sham, and DBS) (Fig. 5B, D, and F correspondingly). Also, the old DBS group (Fig. 5G) had mild neuronal dystrophic changes, while the old control and sham groups (Fig. 5C and E) had severe neuronal dystrophic changes in CA1 region.

Additionally, in the CA3 region, all old groups (control, sham, and DBS) had significantly higher dystrophic score than their corresponding young groups (*p* < 0.001). Furthermore, the young DBS group showed significantly lower score compared to young sham group (*p* < 0.01) and the old DBS group had significantly lower dystrophic damage score than the old control and sham groups (*p* < 0.001) (Fig. 5H). The CA3 region displayed moderate neuronal dystrophic alterations in photomicrographs taken from young groups (control, sham, and DBS) (Fig. 5I, K and M correspondingly). Moreover, the old DBS group (Fig. 5N) had mild neuronal dystrophic changes, while the old control and sham groups (Fig. 5J and L) had severe neuronal dystrophic changes.

The DG revealed identical results, with a noteworthy increase in the dystrophic score in all old groups when compared to their corresponding young groups (control, sham, and DBS) (*p* < 0.001). Additionally, compared to the old control and sham groups, the old DBS group had a significantly lower dystrophic damage score (*p* < 0.001) (Fig. 5O). The DG region displayed minor neuronal dystrophic alterations in photomicrographs taken from young groups (control, sham, and DBS) (Fig. 5P, R, and T, respectively). The old DBS group (Fig. 5U) had moderate neuronal dystrophic changes, while the old control and sham groups (Figs. 5Q and S) had severe neuronal dystrophic changes in the DG region.

### Effect of DBS on the expression of neuroprotective Hsp70 in different hippocampal regions (CA1, CA3, and DG) of young and old rats

**Fig. 6:**
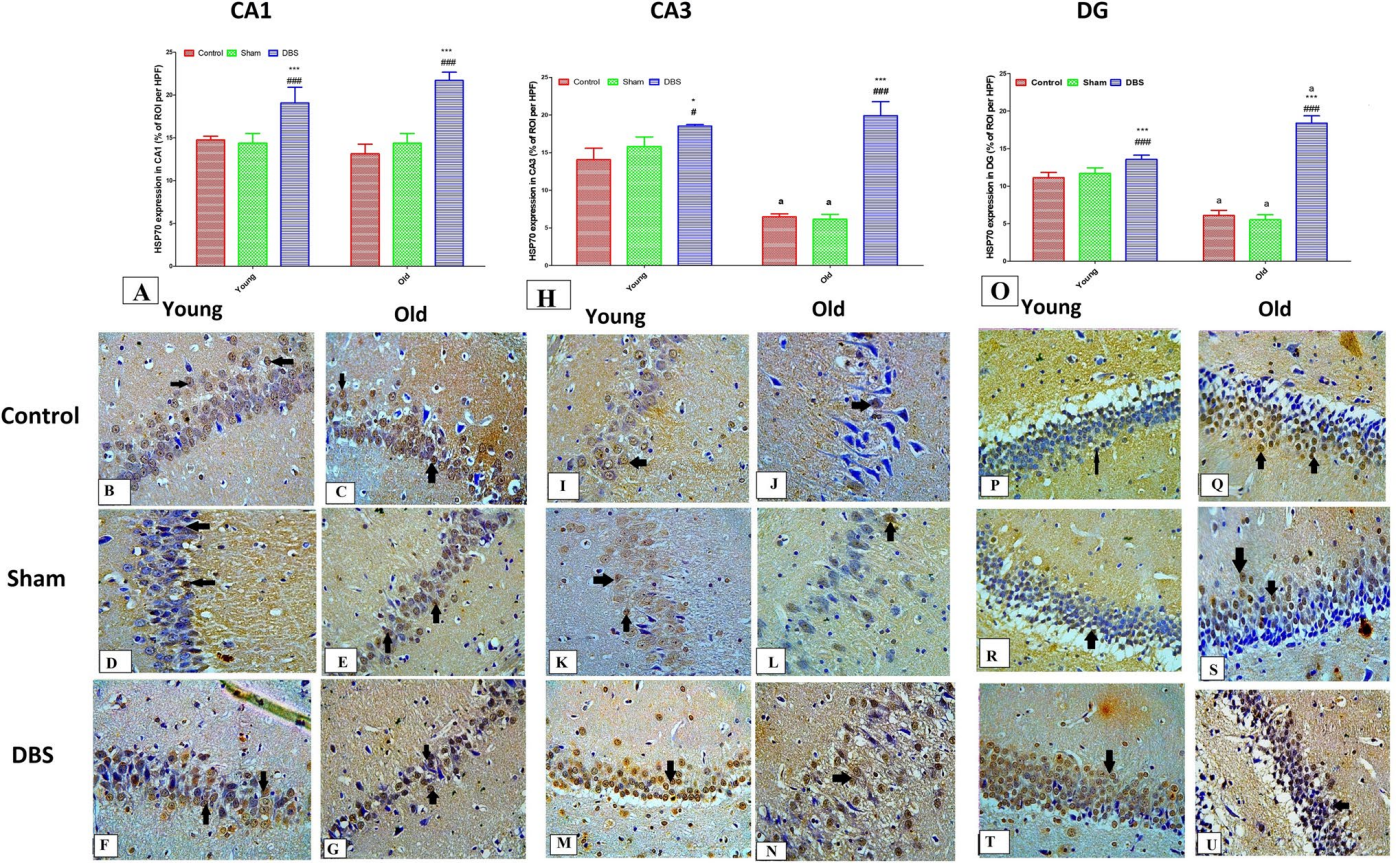
Immunohistochemical examination for Hsp70 in different hippocampal regions (CA1, CA3 and DG) from different groups. Figure A displays the Hsp70 expression score in the CA1 area of hippocampi from various old and young groups. Photomicrographs reveal that the young DBS group (**F**) and the old DBS group (**G**) have high Hsp70 expression, while the young control group (**B**), old control group (**C**), young sham group (**D**), and old sham group (**E**) have moderate neuronal nuclear expression of Hsp70 (brown nuclear staining) (black arrows). Score expression of Hsp70 in the CA3 region is shown in Fig. H. Photomicrographs from the CA3 regions indicate that the expression of Hsp70 is moderate in the young control group (**I**), low in the old control group (**J**), moderate in the young sham group (**K**), low in the old sham group (**L**), high in the young DBS group (**M**), and high in the old DBS group (**N**). The expression score of Hsp70 in the DG region is displayed in Fig. O. Photomicrographs from the DG demonstrate high expression in the young control group (**P**), moderate expression in the old control group (**Q**), high expression in the young sham group (**R**), moderate expression in the old sham group (**S**), high expression in the young DBS group (**T**), and high expression in the old DBS group (**U**). Two-way ANOVA with Tukey post hoc tests were used for comparing different groups of at different age times. ^a^ significant vs the corresponding young group, * significant vs the control group and #significant vs the sham group of the same age. *p < 0.05, ***p < 0.001, ^#^*p* < 0.05,^###^*p* < 0.001. DBS = deep brain stimulation

Hsp70 expression showed no statistically significant differences between all young groups (control, sham and DBS groups) and their corresponding old groups in the CA1 region of hippocampus. Moreover, DBS groups (old and young) showed significant increase in Hsp70 expression compared to their corresponding control and sham groups (*p* < 0.001) (Fig. 6A). On the other hand, in CA3 and DG, the results indicated a substantial decrease in old control and sham groups when compared to their corresponding young groups (*p* < 0.001). Furthermore, compared to their corresponding control and sham groups, DBS group showed significantly higher Hsp70 expression in both the young and old groups (*p* < 0.001) (Fig. 6H and O).

The photomicrographs in Figs. 6B, D, and F depict Hsp70's nuclear and cytoplasmic expression in the CA1 region in young (control, sham, and DBS groups, respectively), while Figs. 6C, E, and G depict Hsp70's nuclear and cytoplasmic expression in the CA1 region in old (control, sham and DBS groups, respectively) photomicrographs. Furthermore, photomicrographs from young (control, sham, and DBS groups) are shown in Figs. 6I, K, and M, respectively, demonstrating nuclear and cytoplasmic expression of Hsp70 in the CA3 region. Similarly, photomicrographs from old (control, sham, and DBS groups, respectively) are shown in Figs. 6J, L, and N. Furthermore, photomicrographs from the young (control, sham, and DBS groups) in Figs. 6P, R, and T demonstrate a nuclear and cytoplasmic expression of Hsp70 in the DG region; photomicrographs from the old (control, sham, and DBS groups) in Figs. 6Q, S, and U demonstrate a nuclear and cytoplasmic expression of Hsp70 in the DG region.

### Effect of DBS on the expression of BDNF in different hippocampal regions (CA1, CA3 and DG) of young and old rats

**Fig. 7:**
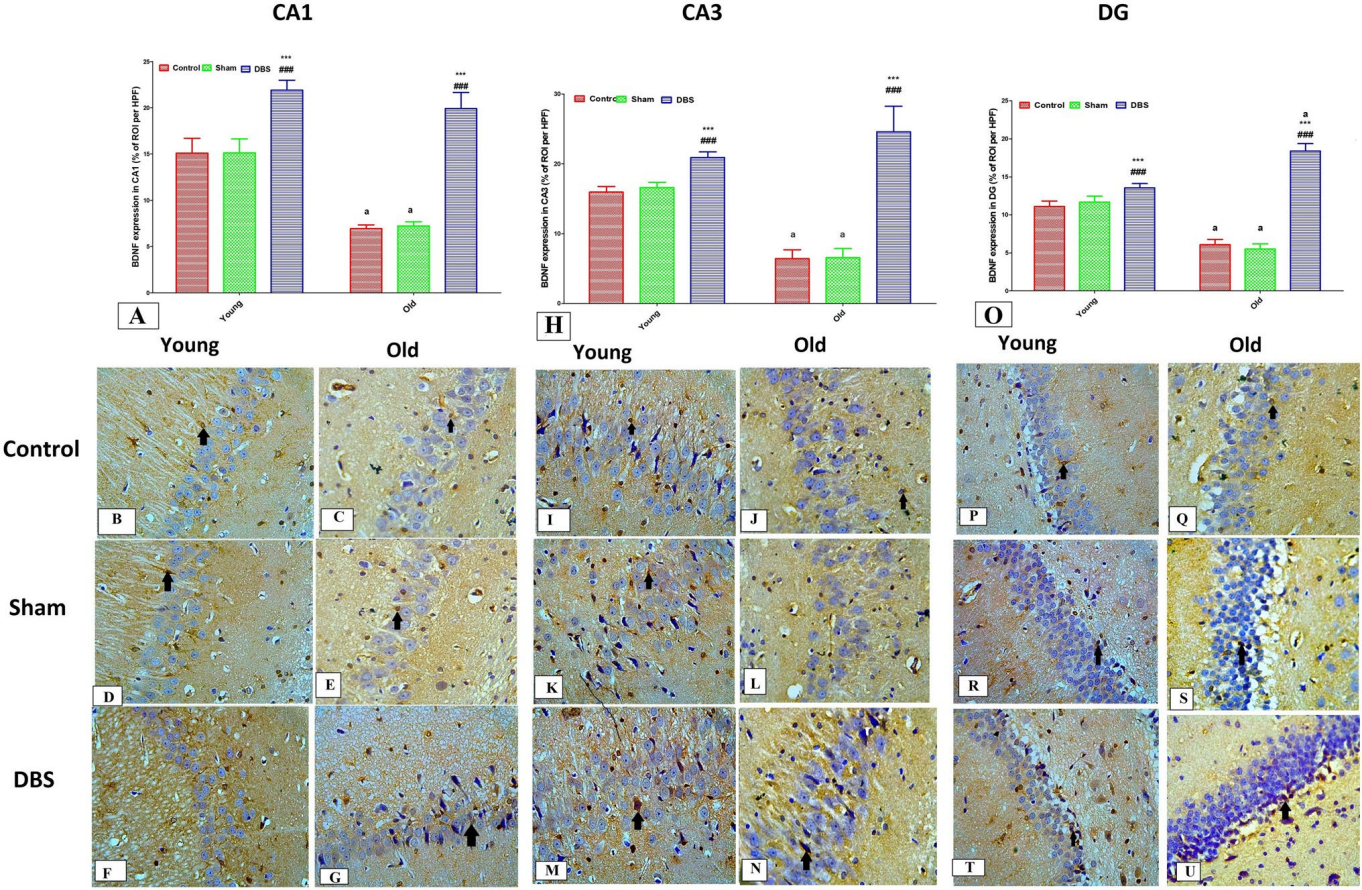
Immunohistochemical examination for BDNF in different hippocampal regions (CA1, CA3 and DG) from different groups. Figure A displays the BNDF expression score in the CA1 area of hippocampi from different old and young groups. Photomicrographs demonstrate that the young control group (**B**) had moderate cytoplasmic and axonal expression of BDNF (brown nuclear staining) (black arrows); the old control group (**C**) had minimal expression; the young sham group (**D**) had moderate expression; the old sham group (**E**) had minimal expression; and the young DBS group (**F**) had high expression of Hsp70 and the old DBS group (**G**) had moderate expression. A photomicrograph from the CA3 regions reveals minimal expression in the young control group (**I**), very low expression in the old control group (**J**), minimal expression in the young sham group (**K**), very low expression in the old sham group (**L**), and moderate expression. Fig. H displays the score of BDNF expression in the CA3 region. Photomicrographs derived from CA3 regions exhibit variable levels of expression: minimal in the young control group (**I**), very low in the old control group (**J**), minimal in the young sham group (**K**), very low in the old sham group (**L**), moderate in the young DBS group (**M**), and moderate in the old DBS group (**N**). The BDNF expression score in the DG area is displayed in Fig. O. According to DG photomicrographs, there is mild expression in the old control group (**Q**), moderate expression in the young control group (**P**), mild expression in the old sham group (**S**), moderate expression in the young DBS group (**T**), and moderate expression in the old DBS group (**U**). Two-way ANOVA with Tukey post hoc tests were used for comparing different groups of at different age times. ^a^ significant vs the corresponding young group, * significant vs the control group and #significant vs the sham group of the same age. *p < 0.05, ***p < 0.001, ^#^*p* < 0.05,^###^*p* < 0.001. *DBS* deep brain stimulation

**Fig. 8:**
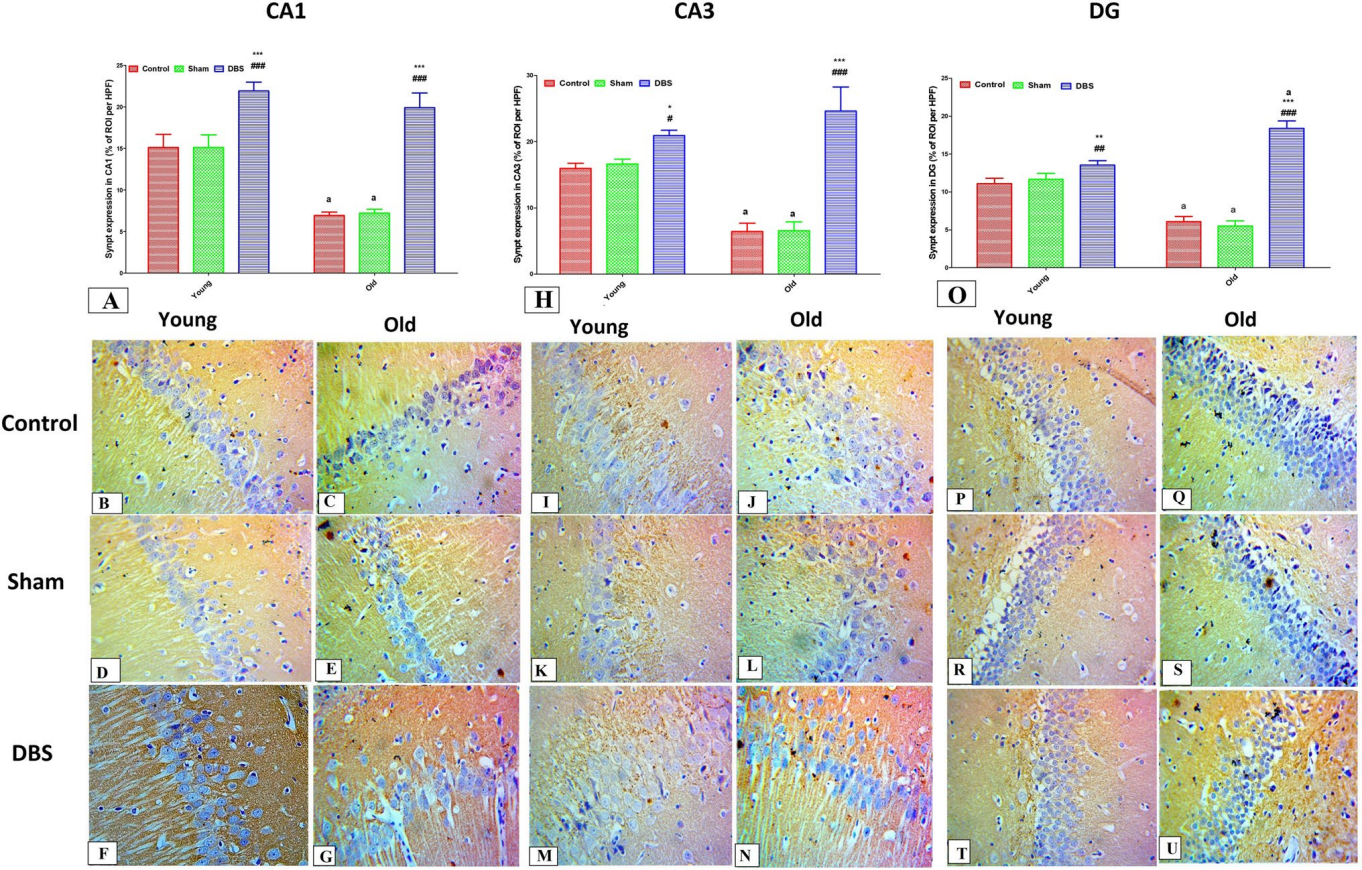
Immunohistochemical examination for synaptophysin in different hippocampal regions (CA1, CA3 and DG) from different groups. The synaptophysin expression score in the CA1 area of hippocampi from various age and young groups is displayed in Figure **A**. Photomicrographs demonstrate high Hsp70 expression in young DBS group (**F**) and moderate expression in old DBS group (**G**), as well as moderate axonal expression of synaptophysin (brown nuclear staining) (black arrows) in young control group (**B**), minimal expression in old control group (**C**), moderate expression in young sham group (**D**), and minimal expression in old sham group (**E**).The expression score of synaptophysin in the CA3 region is displayed in Fig. H. Photomicrographs from the CA3 regions indicate moderate expression in the young control group (**I**), minimal expression in the old control group (**J**), moderate expression in the young sham group (**K**), minimal expression in the old sham group (L), high expression in the young DBS group (**M**) and high expression in the old DBS group (**N**). The expression score of synaptophysin in the DG region is displayed in Fig. O. Photomicrographs from the DG region display moderate expression in the young control group (P), mild in the old control group (**Q**), moderate expression in the young sham group (**R**), mild in the old sham group (**S**), moderate expression in the young DBS group (**T**), and moderate expression in the old DBS group (**U**). Two-way ANOVA with Tukey post hoc tests were used for comparing different groups of at different age times. ^a^ significant vs the corresponding young group, * significant vs the control group and #significant vs the sham group of the same age. *p < 0.05, **p < 0.01, ***p < 0.001, ^#^*p* < 0.05,^##^*p* < 0.01, ^###^*p* < 0.001. *DBS* deep brain stimulation

The expression of BDNF in CA1, CA3, and DG, using immunohistochemistry, revealed that the old control and sham groups had significantly lower levels than the corresponding young groups (*p* < 0.001). Furthermore, DBS groups (both young and old rats) showed significantly higher BDNF expression in the CA1, CA3 and DG regions compared to their corresponding control and sham groups (*p* < 0.001) (*p* < 0.001) (Figs. 7A, H and O). Photomicrographs from the young (control, sham, and DBS groups) in Figs. 8B, D, and F demonstrate the cytoplasmic expression of BDNF in astrocytes and neurons of the CA1 region, whereas photomicrographs from the old (control, sham, and DBS groups) in Figs. 7C, E, and G demonstrate the cytoplasmic expression of BDNF in the CA1 region. Furthermore, photomicrographs from young (control, sham, and DBS groups) are shown in Figs. 7I, K, and M, respectively, demonstrating a cytoplasmic expression of BDNF in the CA3 region, whereas photomicrographs from old (control, sham, and DBS groups, respectively) are shown in Figs. 7J, L, and N. Additionally, photomicrographs from young (control, sham, and DBS groups) in Figs. 7P, R, and T demonstrate a cytoplasmic expression of BDNF in the DG region, whereas photomicrographs from old (control, sham, and DBS groups) in Figs. 7Q, S, and U demonstrate a cytoplasmic expression of BDNF in the DG region.

### Effect of DBS on the expression of synaptophysin in different hippocampal regions (CA1, CA3, and DG) of young and old rats

When comparing the old control and sham groups to their corresponding young groups, an immunohistochemical analysis of the expression of synaptophysin in CA1, CA3, and DG revealed a substantial decrease (*p* < 0.001). Additionally, DBS significantly increased the expression of BDNF in CA1, CA3, and DG when compared to the relevant control and sham groups (*p* < 0.001) in both the young and old groups (Fig. 8A, H and O). Photomicrographs of the CA1 region's neurons from young (control, sham, and DBS groups) and old (control, sham, and DBS groups) respectively, Figs. 8B, D, and F and Fig. 8C, E, and G respectively, demonstrate the cytoplasmic axonal expression of synaptophysin in cells of the CA1 region. Additionally, cytoplasmic axonal expression of synaptophysin in neurons of the CA3 region was seen in photomicrographs 8 J, L, and N from old (control, sham, and DBS groups, respectively) and Figs. 8I, K, and M from young (control, sham, and DBS groups, respectively). Furthermore, photomicrographs of DG region neurons from young (control, sham, and DBS groups) and old (control, sham, and DBS groups) respectively, Figs. 8P, R, and T, and Figs. 8Q, S, and U, respectively, demonstrate the cytoplasmic axonal expression of synaptophysin in cells of the DG region.

### Correlations between different types of memories, LFPs, and molecular biomarkers in hippocampal tissues

The PAT entry latency time exhibited a negative association with the dysmorphic score in the CA 1 and CA3 areas (*p* < 0.01 for both), and a positive correlation with the antioxidants (GSH, Nrf2 and HO-1), Hsp70, BDNF, and synaptophysin expressions. Conversely, the proportion of spontaneous modifications in the Y maze did not exhibit any significant link with any of the parameters that were examined, with the exception of the expression levels of synaptophysin and Hsp70 in the CA1 region (*p* < 0.044). Additionally, in the CA1 and CA3 regions, there were negative connections (*p* < 0.022) between the dystrophic score and the GSH and Nrf2 expression levels and the expressions of Hsp70, BDNF, and synaptophysin. Furthermore, there are negative relationships (*p* < 0.015) between the dystrophic score in CA1 and CA3 and the levels of synaptophysin, BDNF, and Hsp70 expression in those regions (Table 2).

**Table 2:** Correlations between different types memories and LFPs and molecular biomarkers in hippocampal tissues

	PAT	Ymaze	Slow gamma	Fast gamma	MDA	GSH	CAT	Nrf2	HO-1	Hsp70 CA1	Hsp70 CA3	BDNF CA1	BDNF CA3	Syn CA1	Syn CA3	Dys CA1	Dys CA3
PAT	1	.334* .044	-.143 .444	-.086 .684	.306 .069	.455** .005	-.275 .104	.410* .013	.446** .006	.442** .007	.759** .000	.684** .000	.741** .000	.790** .000	.730** .000	-.699** .000	-.747** .000
Ymaze		1	-.072 .725	.008 .972	-.259 .133	.048 .782	-.143 .414	.068 .698	.081 .644	.264 .125	.343* .044	.223 .198	.222 .201	.431* .035	.284 .098	-.205 .238	-.216 .213
Slow gamma			1	-.159 .448	-.164 .443	-.261 .219	-.094 .661	-.201 .346	-.200 .350	-.005 .980	-.184 .379	-.026 .898	-.065 .763	-.236 .267	-.287 .174	.244 .250	.284 .179
Fast gamma				1	-.506* .012	.284 .179	.268 .205	.566** .004	.507* .011	.494* .014	.163 .457	.035 .867	.131 .552	.001 .996	.408* .048	.150 .485	-.037 .865
MDA					1	.198 .247	-.43** .010	.299 .077	.378* .023	.271 .110	.313 .067	.370* .026	.262 .128	-.413* .045	.091 .596	.034 .845	.082 .635
GSH						1	.339* .043	.843** .000	.843** .000	.556** .000	.593** .000	.777** .000	.642** .000	.751** .000	.728** .000	-.396* .017	-.554** .000
CAT							1	.196 .253	.130 .450	-.175 .307	-.450** .007	-.077 .654	-.178 .306	.151 .481	-.145 .397	-.033 .850	.088 .608
Nrf2								1	.968** .000	.645** .000	.633** .000	.697** .000	.584** .000	.787** .000	.852** .000	-.380* .022	-.584** .000
HO-1									1	.264 .125	.343* .044	.223 .198	.222 .201	.713** .000	.853** .000	-.224 .189	-.461^**^ .005
Hsp70 CA1										1	.592** .000	.519** .001	.561** .000	.373 .073	.553** .000	-.046 .792	-.341* .042
Hsp70 CA3											1	.785** .000	.701** .000	.719** .000	.782** .000	-.484** .003	-.687** .000
BDNF CA1												1	.727** .000	.828** .000	.813** .000	-.564** .000	-.652** .000
BDNF CA3													1	.601** .002	.838** .000	-.409* .015	-.631** .000
Syn CA1														1	.728** .000	-.762** .000	-.858** .000
Syn CA3															1	-.451** .006	-.648** .000
Dys CA1																1	.869** .000
Dys CA3																	1

	Before				After				
	Young		Old		Young		Old		
	Sham	DBS	Sham	DBS	Sham	DBS	Sham	DBS	
Delta	49.43 ± 9.38	28.98 ± 9.34	2.33 ± 0.85	20.16 ± 8.13	28.71 ± 9.29	28.03 ± 8.77	7.77 ± 5.15	8.92 ± 5.07	
Theta	5.57 ± 2.41	2.15 ± 1.50	1.89 ± 0.86	0.92 ± 0.26	6.00 ± 2.16	4.15 ± 1.51	2.00 ± 0.75	1.95 ± 0.43	
Alpha	6.71 ± 2.95	2.62 ± 0.87	1.11 ± 0.42	1.17 ± 0.36	5.14 ± 2.28	3.32 ± 0.91	1.55 ± 0.69	2.00 ± 0.56	
Beta	6.14 ± 2.93	2.14 ± 0.38	5.44 ± 1.64	3.00 ± 1.03	6.28 ± 2.90	3.31 ± 0.93	5.22 ± 1.94	5.33 ± 1.32	
Low Gamma	26.85 ± 8.49	51.79 ± 7.57	58.00 ± 5.47	53.17 ± 6.96	37.14 ± 12.33	38.09 ± 7.98	58.78 ± 6.24	52.33 ± 6.38	
High Gamma	9.50 ± 3.89	13.90 ± 5.39	27.67 ± 4.26	23.50 ± 5.67	7.67 ± 1.38	31.24 ± 7.42	26.00 ± 5.13	31.83 ± 4.44	

Pearson’s correlation, **. Correlation is significant at the 0.01 level (2-tailed), *. Correlation is significant at the 0.05 level (2-tailed)

## Discussion

The present study discovered that aged rats' spatial and working memories had severely diminished. This was greatly enhanced by DBS of the LHA using high frequency electric currents for 1.5 h over the course of five days. According to earlier research, DBS for the ventromedial prefrontal cortex (PFC), fornical area, entorhinal cortex, and anterior thalamus significantly improved cognitive abilities and memory in both human and animal studies [45, 49, 78, 50]. On the other hand, a small number of studies have suggested that DBS for the ventromedial PFC and anterior thalamic nuclei impairs memory [28, 96]. The difference in findings could be attributed to DBS's timing. On the other hand, DBS given a few days before the behavioral assessments had positive results. Only when DBS was given during or right after the behavior test did impairments occur [49]. Thus, we postulated that stimulation before a memory test is beneficial because it enhances memory through synaptic plasticity, whereas DBS during or following a memory task interferes with memory consolidation or acquisition. For this reason, we used a DBS protocol in the current study, which involves stimulating the brain for 30 min, followed by 5 min of rest, for three cycles in a single session. Furthermore, Chamorro-López et al. [15] discovered that rats who received intracellular Lucifer yellow post-training self-stimulation of the LHA exhibited enhanced memory in the spatial water maze challenge. This improvement was correlated with a rise in the structural plasticity of hippocampus CA1 neurons.

Albert-Gascó et al. [5] reported that LHA stimulation may directly affect hippocampus neurons, maybe through the septum/diagonal band. Thus, we looked at the morphology of the hippocampal regions in young and old rats that had DBS. Several investigations have established a connection between memory deficits and anatomical changes in the hippocampus, such as neuronal dystrophies [35, 8]. Neuronal degenerations in the CA1 and CA3 regions were observed in old male and female rats [35, 8]. In the current study, we found the same results of neuronal degenerations in elderly rats (24 months) in the dentate gyrus (DG) and both the hippocampal areas (CA1 and CA3). Furthermore, in old and young rats, DBS stimulation significantly reduced the amount of neuronal damage in the CA1, CA3, and DG hippocampus areas, suggesting a neuroprotective function for DBS in aging. The effects of DBS have been attributed to a number of processes, including altering electrophysiological changes, promoting neurogenesis, and releasing neurotransmitters [82]. However, further research is still needed to pinpoint the precise mechanisms and pathways.

The spikes of many neurons or local field potentials (LFPs) represent the electrical activities of these neurons and might reflect mental processes. For instance, in both human and animals, fast gamma waves have been linked to a variety of behavioral situations, including movement, memory, attention, and decision-making. In contrast, slow gamma waves, which may be observed in freely acting rats, have been linked to action and movement [17]. Since the CA1 hippocampal area serves as the principal output of the hippocampus to other brain regions, an analysis of its outputs provides an elegant representation of the overall hippocampal information processing, which is why we recorded LFPs from this region in the current study [97]. Furthermore, the first section of the hippocampus to be impacted by Alzheimer's disease is the CA1 region [97]. Furthermore, phase synchronization in gamma band activity has been shown to be important for memory encoding [55, 21].

In freely moving animals, theta and gamma oscillations are the most noticeable rhythms, according to electrophysiological recordings from the hippocampal regions [12, 51]. But in the present study, we found that gamma and delta oscillations were the most prominent LFP waves in CA1. The difference between our study and the earlier ones may be explained by the fact that theta waves' frequency in the current study was between 4 and 8 Hz, whereas it was between 3 and 12 Hz in the previous studies [98, 52]. Suthana et al. [79] discovered that entorhinal cortex DBS in patients could improve memory by resetting theta oscillations and boosting phase stability in the hippocampus. Theta-gamma coupling has also been shown to be increased by DBS for entorhinal cortex in humans, indicating a potential mechanism through which gamma frequency DBS may function to alter theta frequency oscillations to modulate memory [2]. In the present study, we found that DBS significantly increases the fast gamma waves in both young and old rats, as well as theta waves in old rats. These results imply that enhancing the fast gamma oscillations in the CA1 of the hippocampus could be a potential mechanism for the advantageous effects of DBS on memory decline brought on by aging. Furthermore, since the fornix is closely related to LHA and the forniceal septohippocampal pathway produces theta–gamma cross-frequency coupling and theta and gamma oscillations, which are critical for memory functions, these electrophysiological changes in the current study with DBS may be caused by forniceal affection by this stimulation [72].

One of the main causes of the age-dependent cognitive deterioration is oxidative stress. The brains of elderly animals have higher concentrations of oxidative stress markers and reactive oxygen species (ROS) than those of younger animals [39]. Furthermore, increased oxidative species and stress were highly correlated with cognitive impairment in learning, memory retention, and temporal and spatial memory in animal models of aging [39, 24]. According to this hypothesis, the current study's findings of increased oxidative stress in the hippocampal regions of aged rat control rats compared to young rats were supported by the decrease in GSH and CAT activities in the hippocampal regions of aged control rats. Additionally, we found that antioxidant GSH levels in the hippocampal regions and working memory were positively correlated. Obydah et al., [61] showed a notable increase in oxidative stress in the hippocampus of elderly rats, which is consistent with these findings. Furthermore, Misrani et al., [57] reported that neuronal degeneration and a loss in cognition in Alzheimer's patients have been linked to mitochondrial oxidative stress brought on by mitochondrial damage. Furthermore, Butterfield [11] reported that a significant amount of oxidative damage to the brain was present in the samples of mild cognitive impairment (MCI) patients that preceded Alzheimer's disease. This damage included protein carbonyls, lipid peroxidation, and malondialdehydes. Depleted antioxidant systems may be the ultimate consequence of elevated oxidant species levels, according to a study that also shown a clear link between lower antioxidant levels and higher lipid peroxidation [66].

Additionally, compared to control groups, the current study showed a substantial increase in MDA (lipid peroxidation marker) in the sham groups (old and young), which suggests that the insertion of the recording and stimulating electrodes in the rat's brain increased the redox status in the hippocampus. Furthermore, DBS stimulation in both young and old rats resulted in a significant decrease in the hippocampal MDA level and a significant increase in GSH levels and CAT activities, indicating that LHA deep stimulation may enhance antioxidants and lessen oxidative stress brought on by aging in the hippocampal region. Additionally, the current study showed a significant reduction in the expression of the antioxidant genes HO-1 and Nrf2, which is consistent with earlier research showing significant reductions in the expression of these genes in the hippocampus and spinal cord [18], as well as in the dependent antioxidant gene HO-1 at the levels of mRNAs [61, 46], and proteins [46] in aged rats. These results point to a decrease in Nrf2 expression and activity in the hippocampal regions with aging. Furthermore, following stereotactic surgery in young, sham mice, the expression of Nrf2 and its controlled antioxidant genes, including HO-1, was markedly elevated in the hippocampus. On the other hand, the levels of Nrf2 and its target genes did not significantly alter in response to surgery in old rats. These findings imply that a healthy Nrf2 system in the brain is necessary for Nrf2-regulated antioxidant defense. Downregulation of Nrf2 associated with aging prevents the activation of an antioxidant defense mechanism, making the hippocampal region more susceptible to oxidative stress and neuroinflammation brought on by surgery, which in turn results in cognitive impairment [46]. Our results are consistent with a previous study that found that knockdown of Nrf2 in mice did not change the expression of proinflammatory cytokines in the brain under normal settings or the levels of its downstream target genes. Nevertheless, Nrf2 knockdown eliminated the brain's response to acrylamide by upregulating Nrf2-mediated antioxidant genes, which increased the expression of proinflammatory cytokines and neurotoxicity [20].

Due to ongoing exposure to harmful environmental stimuli, aging is accompanied by a decline in the body's defense mechanisms as well as an accumulation of damage at the molecular, cellular, and organic levels [77, 77]. Heat shock proteins (Hsps), which are vital intracellular proteins in all living things, are one of these defense systems. In Parkinson's disease, Alzheimer's disease, polyglutamine disorders, and amyotrophic lateral sclerosis, Hsps can shield neurons from toxicity and protein aggregation. They can also shield cells from apoptosis in Parkinson's disease and inflammation brought on by cerebral ischemia injury [32, 85]. Numerous experimental illness models, such as the rat model of Parkinson's disease and the β-amyloid model of Alzheimer's disease, have demonstrated the protective function of Hsp70 [7, 37, 42]. One potentially effective treatment approach for these protein structural abnormalities is to induce Hsp70 expression. According to earlier research, neurodegenerative illnesses may benefit from the application of Hsps chemical inducers including PGA1 [89], geldanamycin [74], and valproic acid [92]. Walters et al.,[87], reported that aged rats showed much higher expression levels of Hsp70 when compared to young rats, indicating that the brains of older rats continue to be able to accumulate Hsp70 in response to heat stress. Moreover, Unno et al., [86] found that 24-month-old rats' pons, medulla, striatum, and thalamus had considerably higher Hsp70 expression than did their cerebral cortex and hippocampal regions. This finding raises the possibility that elevated Hsp70 expression associated with aging may inhibit protein denaturation. In addition, Shao et al., [73] discovered that older rats' hippocampi had significantly lower levels of Hsp70 than did younger rats. Furthermore, they discovered that aging may lower the expression level of Hsp70 mRNA, which could be a result of habituation in a long-term stressful environment. According to the current study, aged rats' Hsp70 levels in the CA1, CA3, and DG hippocampus regions were much lower, whereas DBS significantly increased these levels. Furthermore, in the CA1 and CA3 hippocampus areas, there was a significant connection between Hsp70 expression and working memory. These results validate the role of Hsp70 in aging processes and imply that Hsp70.

Several growth factors, like BDNF, activate neurogenesis, one of the proposed pathways for the positive benefits of DBS. BDNF is primarily produced in the brain's glial and neuronal cell bodies before being transferred to the terminals and dendrites of presynaptic connections. BDNF mRNA is found in the cortical, hippocampus, nigral, amygdala, and thalamic regions of the central nervous system [16]. The hippocampal regions with the highest concentration of BDNF mRNA include the nuclei of granule cells in the dentate gyrus, the pyramidal cell layer, the medial part of CA1, and the CA2 are the main locations of hippocampal BDNF expression [31, 63]. Numerous physiological events and pathological insults, including fear conditioning [83], seizures [76], ischemia [91], and consolidation of hippocampus-dependent memory [68, 9], have been frequently related to activity-dependent BDNF transcription and trafficking. The endogenous activity-dependent pool of BDNF is rapidly depleted over prolonged periods of secretion, as demonstrated in long-term potentiation, despite the fact that BDNF is a crucial molecule for the morphological and functional integrity of the hippocampus formation [34, 65, 71]. In these circumstances, it appears that recycling of released BDNF and de novo synthesis from pre-existing BDNF mRNA are essential for supplying threshold amounts of BDNF to sustain long-lasting synaptic alterations underpinning memory. Furthermore, it has been found that the amount of mature BDNF in the rat brain's hippocampal and cerebral cortex was unaffected by age. In the present work, we found that BDNF levels in the CA1 and CA3 hippocampal areas of elderly rats had significantly decreased [14]. In contrast, DBS significantly increased the production of BDNF, particularly in old rats, and this increase was positively correlated with working memory. These results imply that the mechanism by which DBS protects against age-induced memory impairment may involve the overexpression of BDNF. In order to support nerve growth and maturation during developmental stages, control adult synaptic transmission and plasticity, and enhance memory functions, BDNF is essential [19, 58, 25].

Learning-related cellular plasticity mechanisms have been linked to synaptophysin and other synaptic vesicle proteins [59]. Although a drop in synaptophysin has been linked to a decrease in the number of synapses, other factors, such as a decrease in synapses' size, may also be at play [13, 29, 4]. Liu et al., [48] came to the conclusion that decreased expression of caveolin and synaptophysin in the hippocampus causes aging to be accompanied by a decline in cognitive brain functions and synaptic plasticity. Wen Min et al., [99] discovered that as rats age, the expression of synaptophysin steadily declines in the stratum radiatum of the CA3 region of the rat hippocampal region. Furthermore, Ojo et al., [62] discovered a noteworthy decrease in the expression of synaptophysin in the hippocampal regions of elderly rats. Additionally, the hippocampal expression of aged rats exhibited a considerable decrease in synaptophysin expression [58]. Deficits in spatial learning and memory have been linked to age-related changes in CA3 synaptic alterations, which are reflected as a decrease in synaptophysin [3, 81]. The current investigation showed a significant decrease in synaptophysin expression in the DG, CA3, and CA1 hippocampal areas of aged groups, which is consistent with these studies. Furthermore, DBS significantly increased its expression in adult and juvenile rats. Additionally, there were favorable connections between its expression in CA1 and CA3 and working memory, spatial memory, and the expression of BDNF and Hsp70 in the same hippocampal areas. Furthermore, its expression in CA1 and CA3 was positively correlated with working memory, spatial memory, and the expression of BDNF and Hsp70 in the same hippocampal areas. These results imply that one possible mechanism by which DBS protects against age-induced memory impairment may be synaptophysin overexpression.

The current study shows several limitations including unilateral stimulation for LHA not bilateral stimulation for LHA as well as recording the electrical activities from CA1 regions, so future studies with bilateral stimulation for LHA are suggested. Although, we tried to be restricted to LHA with electrode insertions with excluding any rat with misplaced electrode, the fornix may be affected by stimulation of LHA. So further studies using new techniques such as optogenetics and fiber photometry that help us to indicate and localize exactly the site of stimulation and the source of the recorded electrical activities from the CA1 are need for better understanding the electrophysiological changes during aging and after DBS.

## Conclusions

In rats, aging is associated with decline in working and spatial memories which might be due to neurodegeneration of hippocampal neurons that is caused by an increase in oxidative stress with downregulation in antioxidant genes (Nrf2 and HO-1), Hsp70, BDNF and synaptophysin. DBS for LHA improve the memory loss brought on by aging. This may be brought on by the hippocampus's sorting of LFPs (increase in fast gamma) in CA1, reduction of oxidative stress, and upregulation of antioxidant genes (Nrf2 and HO-1), Hsp70, BDNF and synaptophysin in the hippocampus.

## Data Availability

No datasets were generated or analysed during the current study.
